# Community feedback sessions: An adaptation of the community engagement studio model to enhance scalability

**DOI:** 10.1017/cts.2026.10745

**Published:** 2026-05-06

**Authors:** Simone C. Frank, Alicia Bilheimer, Nixola Datta, Mary E. Grewe, Eseohe Aikhuele, Khadeejatul-Kubraa A. Lawal, Michael D. Kappelman

**Affiliations:** 1 North Carolina Translational and Clinical Sciences Institute, University of North Carolina at Chapel Hill, Chapel Hill, NC, USA; 2 Division of Pediatric Gastroenterology, Department of Pediatrics, The University of North Carolina at Chapel Hill School of Medicine, USA

**Keywords:** Community engagement, patient engagement, community engagement studio, community feedback session, clinical and translational science

## Abstract

**Introduction:**

The Community Engagement (CE) Studio is an established approach for integrating community and patient perspectives into research. Despite its widespread implementation, little information is available about how this model has been adapted across institutions to accommodate varying resources and capacities.

**Methods:**

The Patient and Community Engagement in Research (PaCER) Program at UNC-Chapel Hill modified the CE Studio model – with adaptations to recruitment, onboarding, virtual delivery, compensation, and session planning – resulting in the development of a new Community Feedback Session (CFS) service. This adaptation yielded a scalable model that requires fewer resources, supports capacity-building within research teams, and leverages technology. We then evaluated the CFS approach via surveys distributed to CFS attendees and researchers.

**Results:**

Between January 2022 and August 2025, we conducted 46 CFSs across 19 projects engaging 302 attendees; 149 attendees and 34 researchers completed evaluation surveys. Of these, 99% of attendees indicated they would participate again, and 97% of researchers rated the CFS service as Good or Excellent and recommended it to colleagues. Respondents appreciated the structure, facilitation, composition, and size of the sessions, and many had no suggestions for improvement. Researchers described changes they made to projects based on feedback received during CFSs, including amending study protocols, altering intervention content and delivery modalities, and revising a variety of study-related materials.

**Conclusions:**

Evaluation data support the acceptability, feasibility, and utility of the CFS approach, indicating that this modified and scalable service accomplishes researcher goals, informs research in an impactful way, and provides value to partners.

## Introduction

It is widely recognized that engaging community and patient partners (partners) in the design, implementation, and dissemination of research yields studies that are more effective and relevant to the populations they aim to impact [1–7]. In turn, many researchers are eager to incorporate engagement methods into their studies, and sponsors now emphasize community engagement (CE) as a key component of the research they fund [1,8,9]. Community Based Participatory Research – or CBPR – is a gold standard engagement methodology that centers a collaborative approach where partners and academic researchers collaborate to conduct research with shared responsibility and decision-making throughout the research process [10–12]. While valuable, CBPR requires a high degree of training and skill, as well as significant time and financial resources. Many clinical and translational researchers do not receive formal training in engagement and lack experience engaging partners, making methodologies like CBPR hard to implement [13,14]. CBPR is also not always an appropriate or feasible approach for partners who are interested in engagement but prefer more flexible options that align with their needs, experience, and bandwidth [15,16]. As such, many investigators seek accessible engagement methods that can be implemented with limited capacity (e.g., limited funds dedicated to engagement, short project timelines) and skillsets (e.g., investigators who are new to engagement or have received no formal training).

One established lower-touch approach for integrating partner perspectives into research is the CE Studio, which facilitates structured discussions between researchers and community members [17,18]. Developed by the Meharry-Vanderbilt Community Engaged Research Core (MV-CERC) in 2009, the CE Studio is a consultative model that allows researchers to obtain project-specific input from patients, community members, and other interest-holders [17,18]. CE Studios can be used in all phases of the research process to increase the relevance or patient-centeredness of a project, respond to funder requirements for community input, or address challenges related to project implementation. Those involved in CE Studios – including researchers, partners, and CE Studio program staff – have described the model as valuable, feasible, and accessible, and have agreed that it improves the overall quality of research [18]. Because of its widespread success and relative reproducibility, the CE Studio was named a “promising practice” for creating infrastructure to support community-partnered research in the 2024 Principles of Community Engagement, 3^rd^ Edition, which called for academic and other institutions to consider using the model to expand their capacity to conduct community-engaged research [1].

CE Studios have been used across various populations and projects, with over 40 institutions in the United States having implemented the model to date [19]. Not only is the CE Studio model ripe for expansion across academic research institutions, but opportunities exist to adapt the model to improve efficiency, minimize resource utilization, promote scalability, leverage web-based technology, and build researchers’ and community partners’ engagement capacity. Despite this, little information is available about how other institutions have modified or tailored the model, or how factors that differ across organizations (e.g., funding, administrative and regulatory processes, staff capacity, etc.) impact translation of the model across various contexts. As the CE Studio model continues to proliferate across academic research centers, it is important to describe how it has been adapted across institutions to suit organizational contexts, resources, and needs, and to assess the feasibility, acceptability, and utility of such adaptations.

In 2022, the Patient and Community Engagement in Research (PaCER) Program at the North Carolina Translational and Clinical Sciences (NC TraCS) Institute – the hub of the National Center for Advancing Translational Science’s Clinical and Translational Science Award (CTSA) at the University of North Carolina at Chapel Hill (UNC-CH) – developed and implemented an adapted version of the CE Studio model, called Community Feedback Sessions (CFS). The goals of this adaptation were to create an approach that: (1) requires fewer programmatic and human resources to maintain over time; (2) supports capacity-building within research teams for conducting future engagement without reliance on PaCER involvement; and (3) is scalable to other institutions with capacity similar to our own. This paper describes our process of adapting the CE Studio model, implementing a CFS service at our Institute, and evaluating the feasibility, acceptability, and utility of the CFS approach.

## Methods

### Background

NC TraCS’s PaCER Program promotes engaged research at UNC-CH by offering engagement guidance, training, and technical assistance to the university’s clinical and translational research community, as well as to patient and community partners across North Carolina [20]. Via conversations with CE staff at other CTSAs and searches of the literature, we identified the CE Studio model as a possible approach to address increased researcher demand for support in implementing accessible engagement methods within their projects. As we assessed our capacity to incorporate CE Studios as a programmatic service offering, we determined the need to alter several process points in the model to facilitate implementation at UNC-CH. This resulted in the development of our CFS service – a modified CE Studio program with adaptations to recruitment, onboarding, virtual delivery, compensation, and session planning processes.

### Overview of the community feedback session approach

**Figure 1.**
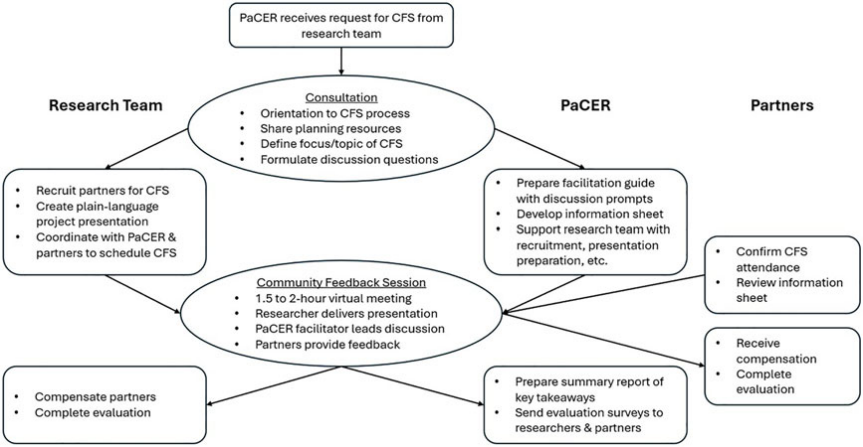
Figure 1 long description. The process for requesting and implementing a Community Feedback Session (CFS) with the Patient and Community Engagement in Research (PaCER) Program.

A CFS provides a structured forum to help researchers gather actionable, authentic, and constructive feedback on their projects from partners, who may be community members, patients, caregivers, healthcare providers, or other interest-holders, depending on the research topic. A CFS includes: (1) consultation with PaCER staff (who have educational backgrounds and training in CE, facilitation, and qualitative methods) to orient a researcher to the CFS process and define the focus of the session; (2) researcher-led recruitment of 4–8 partners who represent the researcher’s population of interest, with guidance from PaCER staff throughout the recruitment process; (3) preparation of session materials, including a facilitation guide with discussion prompts (developed by PaCER staff) and a brief, plain language project presentation (developed by the researcher with input from PaCER staff); (4) a 1-page information sheet (developed by PaCER staff) shared with partners in advance to help prepare for the session; (5) a 1.5- to 2-hour virtual meeting (requests for in-person meetings are considered on a case-by-case basis) that involves delivery of the brief presentation by the researcher, a handful of key questions posed to partners, and discussion guided by a neutral, trained PaCER facilitator to elicit feedback; (6) compensation of partners by the research team; (7) preparation of a post-session summary report of key takeaways (developed by PaCER staff); and (8) session and service evaluations completed by partners and researchers, respectively. This process is outlined in Figure 1. Furthermore, Table 1 outlines critical modifications (discussed in more detail below) in our CFS process compared to the CE Studio model as it relates to the responsibilities of the research team and PaCER staff.

**Table 1.** Comparison table of adaptations to the CE studio model

Community engagement (CE) Studio^21^	Community feedback session (CFS)
*Research team responsibilities:* Meet with CE Studio team to clarify focus of studio, determine characteristics of community expert panel, and formulate discussion questions Receive coaching from CE Studio team on communicating with non-researchers Create and deliver a brief, community-friendly presentation Complete post-CE studio evaluation survey *CE studio team responsibilities:* Meet with research team to clarify focus of studio, determine characteristics of community expert panel, and formulate discussion questions Provide coaching to research team on communicating with non-researchers Recruit community experts Coordinate CE Studio scheduling/logistics Lead 1-on-1 or small group orientation meetings with community experts Facilitate and note-take during CE Studio Prepare written summary of feedback Deliver compensation to community experts Collect evaluations from research team and community experts	*Research team responsibilities:* Meet with PaCER team to be oriented to the CFS process and define focus of session Lead recruitment of community partners to participate in the CFS Coordinate CFS scheduling/logistics in collaboration with PaCER team Create and deliver a brief, community-friendly presentation Provide compensation to CFS attendees Complete post-session evaluation survey and follow-up evaluation surveys *PaCER team responsibilities:* Meet with research team to provide an orientation to the CFS process and define focus of session Provide ongoing consultation to the research team as they prepare for the CFS, including review of recruitment materials and the brief presentation Provide guidance/mentorship to support researcher-led recruitment efforts Provide advice on regulatory and payment processes Collaborate with research team on CFS scheduling/logistics Develop discussion questions, facilitation guide, and 1-page information sheet Facilitate and note-take during CFS Prepare summary report of key takeaways Collect evaluations from research team and CFS attendees

### Key adaptations to the CE studio model

#### Researcher-led recruitment

Unlike the CE Studio model, where CE Studio program staff are typically responsible for recruitment, the CFS service relies on the research teams it supports to recruit their own partners. PaCER staff are available to mentor research teams throughout the recruitment process, providing guidance and best practices; however, PaCER staff are not involved in the recruitment process itself. As CFSs are often geared toward a specific population (e.g., patients with a particular disease), we have found that research teams are more likely than PaCER staff to have expertise about and connections to the specific communities on which their work focuses. To support research teams in their recruitment efforts, PaCER staff offer to: (1) review and revise recruitment materials; (2) distribute recruitment messages to PaCER’s network of partners; and (3) advise research teams on how to include a representative group of people and perspectives in the CFS, though research teams are ultimately responsible for reviewing recruitment screener responses, collecting and storing demographic information, selecting partners, and inviting them to participate in the session.

#### Streamlined onboarding for CFS participants

As part of the CE Studio model, individual or small group onboarding meetings are held with partners prior to participating in a studio [17,21]. This formal orientation led by CE Studio staff involves review of a Community Expert Orientation Guide containing a “community expert” job description, frequently asked questions about the CE Studio process, glossary of common research terms, and forms used during the meeting [21]. In contrast to this tailored, individual orientation, in the CFS model PaCER staff develop a 1-page information sheet to be shared via email, by research teams, with partners a few days prior to a session. This resource contains essential information about session logistics, the research project to be discussed, purpose of the CFS, how to prepare for the discussion (including links to any materials to be reviewed in advance and a selection of the questions to be discussed), and what to expect during and after the session. This approach to onboarding requires minimal time and human resources from PaCER staff and partners, while still adequately preparing attendees for the session.

#### Expedited compensation process

CE Studio program staff typically manage the disbursement of payments to partners [17,19,21,22]. In our CFS model, PaCER staff rely on research teams to distribute payments to their partners using expedited payment processes that our team has developed [23]. This approach reduces the administrative burden placed on PaCER staff, while allowing research teams to oversee and track the disbursement of their own funding to partners. PaCER staff are available to provide guidance throughout the process to ensure that CFS attendees are compensated at a rate commensurate with the expertise and perspective they bring as partners.

#### Virtual CFSs and utilization of technological tools

Core to the original CE Studio model is in-person delivery of sessions. Since the onset of the COVID-19 pandemic, virtual CE Studios have become more common [19,22]. While in-person engagement is valuable and can enhance communication and relationship-building, virtual engagement reduces the amount of time that partners devote to participation, reduces costs associated with in-person attendance (e.g., venue fees, food, mileage reimbursement, childcare), and alleviates other accessibility-related barriers [19,22]. As such, our CFS service centers on the virtual delivery of sessions via the videoconferencing platform Zoom, which can be accessed via the Web or a dial-in phone number. Conducting CFSs virtually has also allowed for the utilization of technological tools to facilitate engagement (e.g., live polling, whiteboarding, chat function) and documentation (e.g., enabling Zoom AI Companion to assist with notetaking) during the session. To address potential challenges related to technological access, digital literacy, and virtual platform familiarity, dial-in options are provided, additional support (e.g., step-by-step instructions, troubleshooting sessions) is available in advance of a session, and requests for in-person session delivery are considered on a case-by-case basis. Measures are also taken to protect partner privacy (e.g., session recordings and notes are stored on a secure server and deleted from Zoom accounts upon project completion, names are removed from summary documents, partners may opt out of participation or participate off-camera).

#### Modified staffing structure to support service delivery

The adapted process points described above have allowed PaCER to streamline the amount and type of human resources dedicated to the CFS service. While the CE Studio model typically relies on multiple distinct staffing roles (i.e., Community Navigator, Facilitator, Scribe, Community-Engaged Research Specialist) [19,21,22], CFSs usually involve 2 PaCER staff – one serving as project lead, with the other serving in a support role. The CFS project lead is responsible for all tasks related to project administration, communication with the research team, material development, session facilitation, summary report preparation, and evaluation. The CFS support role assists with notetaking and technical issues during the session, provides feedback on session materials as needed, and serves as an additional staff contact for the research team. The estimated amount of total PaCER staff time required for conducting a CFS is 16 hours (8 hours for session preparation, up to 4 hours for the facilitator’s and notetaker’s attendance at the session, 4 hours for summary report preparation). Research teams can expect to spend 8–15 hours engaging in CFS-related activities, with total time commitment depending on factors such as session topic and recruitment methods. This differs from the estimated time required to implement a CE Studio, which can be up to 35 hours for the CE Studio Team and 4 hours for a researcher [17,21]. Average time from CFS request to session implementation is ∼4 weeks, though turnaround time varies according to scheduling parameters, recruitment methods, and complexity of session topic. Notably, the CFS service operates on a recharge model, where researchers are expected to provide funds to support PaCER service costs. Our ability to reduce the amount of PaCER staff involved in each CFS has positive budget implications for study teams, allowing the service to remain affordable for those with limited budgets.

#### Planning resources

**Figure 2.**
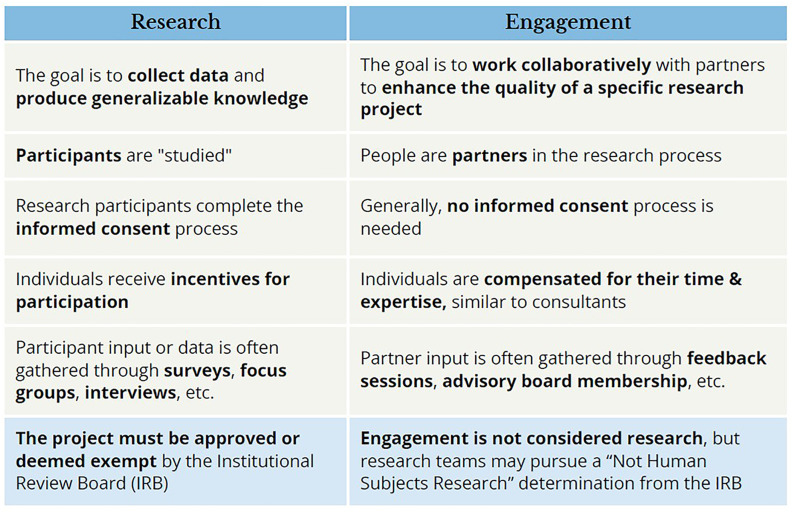
Figure 2 long description. Key differentiations between human subjects research (HSR) and engagement activities as not human subjects research (NHSR).

**Figure 3.**
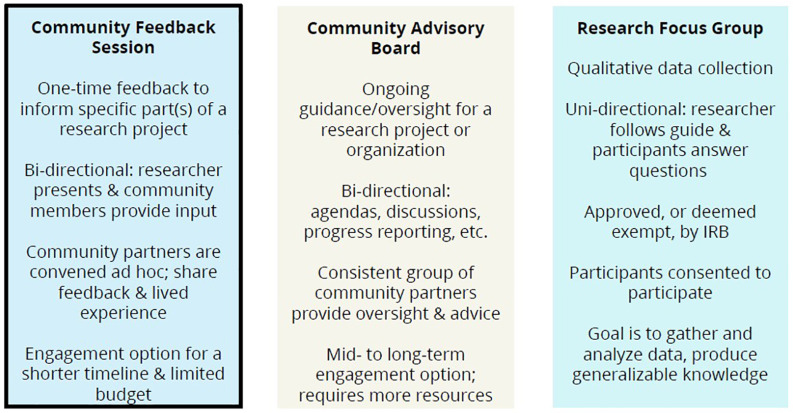
Figure 3 long description. Side-by-side comparison of Community Feedback Sessions, Community Advisory Boards, and Research Focus Groups.

To support early-stage conversations with researchers interested in utilizing the CFS service, PaCER developed a suite of materials to provide concise information about the CFS process, discuss benefits and limitations of the CFS approach, determine if the research team has interest in and capacity to conduct CFS-related activities, and develop a mutual understanding of the purpose and goals of the session (Supplementary Materials 1–5). These include: (1) an overview flyer that outlines the CFS method, process, and frequently asked questions; (2) a slide deck that details how a CFS differs from other engagement and research methods, benefits of the CFS approach, expectations of PaCER staff and research teams when collaborating on a CFS project, a detailed timeline of all CFS-related activities, tips for conducting recruitment, and administrative and budgeting information; (3) a fillable worksheet that serves as a collaborative planning tool and asks research teams to reflect on questions related to session logistics, characteristics of session attendees, plans for recruiting partners, purpose and goals of the session, and envisioned application of feedback gathered, and (4) an Institutional Review Board (IRB) guidance document that describes the differences between human subjects research and engagement and outlines considerations for understanding engagement activities as “Not Human Subjects Research” (NHSR) (to note: PaCER has pursued and received a NHSR designation from UNC-Chapel Hill’s IRB for the CFS service; key differentiations between research and engagement and a side-by-side comparison of the CFS approach to other methods are highlighted in Figures 2 and 3, respectively). Additionally, when a CFS is completed, PaCER requests permission from the research team to share certain materials (e.g., recruitment materials, brief presentation) with future teams who are conducting CFSs on similar topics or are at a similar stage in the research lifecycle. Through this process, PaCER has created a repository of sharable materials that aid research teams in the development of screeners, recruitment flyers and emails, and project presentations. These materials enable research teams to view examples of key documents and tailor them to enhance their relevance and applicability to their own CFS project.

### Evaluation

To facilitate continuous quality improvement of the CFS service and track its related outcomes and impact, PaCER developed a comprehensive evaluation approach, which includes: (1) anonymous post-session surveys to assess researcher and partner experiences and satisfaction with the CFS process, as well as partner demographics; (2) researcher follow-up surveys delivered to one “key contact” on the study team 6–12 months post-CFS to assess how feedback was applied to their research study and any related outcomes; and 3) ongoing written and verbal check-ins with researchers (concluding at 18–24 months) to assess long-term impacts of the CFS. All evaluation surveys (Supplementary Materials 6–8) contain a mix of open-ended, 5-point Likert scale, and multiple-choice questions and were informed by evaluation examples shared in MV-CERC’s Community Engagement Studio Toolkit 2.0 [21]. The intent of the surveys is to evaluate the CFS service, not to measure specific constructs; therefore, no psychometric testing was conducted. Our programmatic evaluation also includes hosting periodic data parties [24,25] – a participatory evaluation event – during which PaCER staff collectively review and interpret evaluation responses and propose changes to CFS service process points to ensure continuous enhancement of service quality and utility. Quantitative survey responses are analyzed via a descriptive statistics approach, while open-ended survey responses are organized into thematic categories. We utilized an available case analysis approach to address missing survey responses [26].

To determine *initial* feasibility, acceptability, and utility of the CFS service, we conducted a brief pilot phase (from January – March 2022), during which PaCER implemented 7 CFSs for 2 research projects (4 CFSs for one project, 3 CFSs for the other) and followed the initial post-session evaluation protocol outlined above. We then held a data party, during which PaCER staff reviewed feedback, identified suggestions for improvement, and proposed changes to the CFS process based on evaluation responses. Resulting recommendations from this event were used to improve the CFS approach prior to our scaling up of the service.

## Results

### CFS characteristics

Between January 2022 and August 2025, we conducted 46 CFSs across 19 projects engaging 302 partners, with an average number of 7 attendees per session. The number of CFSs conducted for any one project ranged from 1 to 5. Common reasons for implementing multiple CFSs within a single project included holding separate sessions: (1) for distinct groups of partners (e.g., clinicians, patients); (2) focused on different topics (e.g., recruitment, intervention development); and 3) at various timepoints across the project lifecycle (e.g., proposal development, study design, dissemination).

The projects for which CFSs were conducted represent a broad range of health topics (e.g., a variety of chronic health diseases, health needs of various populations, etc.) and types of research, including randomized controlled trials, comparative effectiveness studies, and observational studies. Researchers utilizing the CFS service represent an array of academic ranks and disciplines. CFSs were implemented at various stages of the research process, and focused on a variety of topics, including: motivators for and concerns about participating in a particular study; barriers to study participation; proposal development; study design and/or intervention development (including discussion of delivery, methods, outcomes, eligibility criteria, etc.); outreach and recruitment approaches; review of study-specific materials (e.g., logos, flyers, websites, infographics, social media ads, recruitment scripts, consent forms); participant engagement and retention; and strategies for dissemination, communication, and sharing of study results. Table 2 highlights the characteristics of a sample of completed CFSs.

**Table 2.** Characteristics of and selected outcomes from a sample of community feedback sessions (CFS)

Research topic	Investigator department	CFS attendees	Focus of CFS	Sample CFS questions	Sample feedback	Sample outcomes
Comparative effectiveness of inflammatory bowel disease (IBD) treatments	Pediatric gastroenterology	Nurses, physicians, parents of children with IBD, young adults with IBD	Proposal development	How relevant is this study topic? What study design is most appropriate / feasible? What outcomes are most important to measure and at what timepoints? What factors might affect someone’s choice of treatment?	This research topic is of high priority to the pediatric IBD community Prospective cohort is the most appropriate design; it will produce dependable results for patients and providers to inform decision-making A patient-reported outcome (PRO) should be the primary or secondary outcome	Importance of research topic to IBD community noted in proposal Prospective cohort chosen as study design PRO chosen as co-primary outcome Grant submitted and funded
Intervention to reduce diabetes distress	Nutrition	Adults with type 1 diabetes	Intervention development	What is the preferred timing / structure for group intervention sessions? How can the clarity / relevance of weekly activities be improved? How useful are patient data examples? What can make the intervention content more approachable or relatable?	Evening sessions are most accessible, text message reminders and calendar holds are helpful Provide clear, plain-language instructions for intervention activities Providing more information to contextualize example data would make content more engaging / relatable Consider simpler options for activities to minimize challenges with technology, like live polling in Zoom	Intervention delivery modalities and content modified Frequency and mode of communication with participants updated Series of study overview documents developed
Genetic and environmental causes of extremely picky eating	Psychiatry and nutrition	Parents of children with avoidant-restrictive food intake disorder (ARFID)	Recruitment	What would make you / your child want or not want to participate? Would you share information about this study with others? Why or why not? How can the study’s recruitment goal best be reached? How could recruitment materials (study logo, social media ads) be improved?	Helping families and increasing awareness / knowledge about ARFID are motivators for participation Consider recruiting via parent Facebook groups and pediatric providers Recruitment messages should appeal to lived experiences of parenting children with ARFID Recruitment images should be child-centered and capture the impact of ARFID on quality of life	Study logo, website, and recruitment questionnaire redesigned Recruitment goal met ahead of schedule
Effects of estrogen treatment on symptoms of perimenopause	Psychiatry	Individuals who experienced or are experiencing perimenopause	Study design, Recruitment	What would you like to see done differently in terms of how this study is designed? How appropriate / relevant are the recruitment materials to your community? How can the research team better share information about the study?	Address issues related to mistrust, stigma, and restrictive exclusion criteria to make the study more accessible Simplify recruitment messaging; use bullet points, replace scientific jargon with recognizable symptoms, use images of real people Expand recruitment to community-based organizations	Medical exclusion criteria for study participation loosened after meeting with study physician Recruitment materials revised Feedback incorporated into subsequent grant proposals, which received funding
Behavioral intervention to enhance parent–child relationships	General pediatrics	Parents / caregivers of children 18 months-6 years who discontinued study participation	Participant retention	What were your impressions of the initial group session(s)? What were your reasons for not continuing with the sessions? What would have helped you, or made you decide, to continue participating in the study?	Re-structure in-session activities to foster more interaction between participants; rearrange the take-home activities Allow those who miss sessions to join another group or attend make-up sessions Share a program outline with participants at the study’s start to better outline expectations	Video recording take-home activity moved to a later session Flexible rescheduling / make-up policy for missed sessions incorporated Additional, study team-led CFSs conducted with interventionalists
Longitudinal study of children born at least 3 months early	Neonatal-perinatal medicine	Young adults who were extremely low gestational age newborns (ELGANs) and parents of ELGANs	Participant engagement, Research agenda development	Are you interested in connecting with other participants? In what ways? How would you like research results to be shared with you? What format(s) do you prefer? What topics should future research studies focus on?	Connecting to those with similar experiences, either online or in-person, is valuable Results could be shared via newsletter, online portal, email, and/or video / social media Interpersonal relationships, mental health support, and healthy environment are important things to consider for future research on wellbeing	Participant engagement coordinator hired Participant and Family Advisory Board developed to advise study engagement strategies and future directions
Enhancing patient education and decision-making through multimedia	Gynecologic oncology	Patients who have or have had gynecologic cancer or other cancers	Dissemination	What makes an educational resource useful or impactful to you? What are your reactions to the educational videos? How could they be improved? What are ways patients and families can be involved in the creation of new educational materials?	Plain language explanations of the medical procedure and patient / family stories are impactful Use eye-catching graphics and bullet points, avoid wordiness, consider making videos available in multiple languages Supporting materials, like a handout to take notes on or list of questions to ask the provider, could supplement the videos	Feedback incorporated into new set of educational videos Videos translated into Spanish Partnership developed with patients and ovarian cancer advocacy organization to help create future educational content and tools
Social media-based intervention to reduce social isolation and loneliness	School of journalism and media	Young adults aged 18-29	Dissemination, Real-world implementation	Who would you want to see posting these kinds of messages? Where and how would you want to see these messages posted? Which elements of the posts can be changed to better resonate with people in different settings?	Posts from peers, influencers, and special interest groups on platforms like TikTok, Instagram, and Reddit would be most impactful Use engaging formats (e.g., story times, get ready with me, motivational videos) to capture attention Behavioral tips, visual social cues, and stories should be customizable	Editable formats of intervention messages created Instructions for adapting and using the intervention developed

### CFS attendee evaluation

Post-session evaluation surveys were sent to all partners who attended a CFS. The overall response rate was 49%; however, respondents were able to skip questions which resulted in varied response rates by item. Partners who completed the post-session evaluation survey (*n* = 149) were diverse in terms of race and ethnicity, age, and gender (Table 3). Respondents reported an overwhelmingly positive experience participating in CFSs. Almost all (95%, *n* = 125) partners reported that the CFS was worth their time, and 99% (*n* = 148) indicated that they would be willing to participate again; 94% (*n* = 124) said they felt comfortable sharing their thoughts and ideas during the session, and 95% (*n* = 124) agreed that the researcher’s presentation gave them enough information to provide appropriate feedback. Partners endorsed contributing to research projects in a variety of ways during the sessions, including increasing researcher understanding of their point of view/experiences (77%, *n* = 99); sharing ideas on how to inform patients, providers, or community members about the project (64%, *n* = 82); sharing ideas on recruiting research participants (60%, *n* = 78); providing feedback on the importance of the study topic (56%, *n* = 72); providing feedback on how practical or doable the project would be (50%, *n* = 64); and sharing ideas on how to use project results to benefit the community (33%, *n* = 42).

**Table 3.** Demographics of CFS attendees who completed the post-session evaluation survey (*n* = 149)

Demographic category	Response category	*N* (%)
Race	White	66 (54)
	Black / African American	34 (28)
	Asian or Pacific Islander	8 (7)
	American Indian / Alaska Native	5 (4)
	Prefer to self-describe	11 (9)
Hispanic or Latino/a	Yes	25 (19)
	No	106 (81)
Age (Years)	18–24	8 (6)
	25–34	28 (21)
	35–44	27 (21)
	45–54	29 (22)
	55–64	23 (18)
	65 and older	16 (12)
Gender	Woman	100 (78)
	Man	25 (19)
	Non-binary	4 (3)

The evaluation survey also included open-ended questions that explored what partners liked about participating in the session, how it could be improved, and how it affected their feelings about or understanding of research. Respondents shared that they liked the structure, organization, facilitation, and size of the sessions; appreciated sharing their opinions and experiences in an encouraging environment; liked connecting and interacting with a diverse group of other partners; and enjoyed learning more about current research within their areas of interest. With respect to session delivery, partners shared positive comments related to the virtual format of meetings, utilization of virtual tools (e.g., live polling, chat function, whiteboarding), and ability to interact with study-related materials (such as websites and videos) in real time on Zoom. Several partners noted in open-ended responses that they found receiving information via email in advance of sessions to be efficient and effective. Many respondents had no recommendations for improving future sessions; recommendations received were primarily related to session logistics. Partners shared that the sessions emphasized the importance of research and broadened their perspectives about different ways of studying health topics; reinforced the value of engagement; sparked feelings of curiosity, confidence, excitement, trust, and interest in the research area; and provided insight into common challenges, barriers, and other experiences that researchers and institutions encounter throughout the research process. Common themes and illustrative excerpts from open-ended CFS attendee evaluation survey responses are summarized in Table 4.

**Table 4.** Common themes from CFS attendee post-session evaluation survey responses

Question	Response themes	Sample responses
What did you like about participating in the feedback session?	Manageable group size Facilitation approaches that encouraged engagement Well-organized session structure Informative preparation materials Convenience of virtual delivery Having diverse perspectives represented in the group Contributing ideas that will benefit the community and improve the relevance of the research Connecting/interacting with other session attendees Learning more about current research	*The group size was perfect, large enough to have diversity of thought and for people to feel comfortable sharing their experiences.* *I felt like there was real investment in listening to the patient voice. The only way that we are going to solve the issue of disproportionate enrollment in clinical trials is to honestly and wholeheartedly collaborate.* *It was cool to see the ins and outs of how this study is being developed. It was fascinating to see it being broken down step-by-step and the conversations on participant engagement.* *The questions were well organized and pertinent. It felt great to do something with all my thoughts on this matter…it was also helpful to have the questions prior to think over before the session.* *I enjoyed the Zoom format with the facilitator. It really helped keep the discussion moving.*
How can future feedback sessions be improved?	Alter length of the sessions (mixed feedback, with some preferring shorter and others longer) Alter size of sessions Enhancements to virtual delivery and facilitation No suggestions for improvement	*Honestly, I thought this was run very well and I don’t have any ideas for improvement.* *Although it went quite smooth and was wonderful, it would be the time commitment. I think an hour is more reasonable.* *Maybe a larger group of people.* *I think mandatory camera use for participants is important. It makes it feel more mutual.* *You might want to send additional literature to prepare for the session.*
In what ways, if any, did the feedback session affect your feelings about or understanding of research?	Emphasized the importance of research Broadened perspectives about different ways of studying health topics Reinforced the value of patient/community engagement Sparked feelings of curiosity, confidence, excitement, trust, and interest in the research area Provided insight into challenges, barriers, and other experiences that researchers encounter	*It reminded me of how important research truly is. When you read statistics or analytical results, we sometimes forget about the real people who are pioneering these studies to help everyone. It made me grateful that there are smart people willing to put in the countless hours behind each study from grant writing to execution to follow up.* *The feedback session gave me a more positive thought about research. It transitioned research from just a number and feeling of being a lab rat. It put a face behind research.* *It gave me confidence in research and a basic understanding of the complexity of doing good research.* *It helped me recognize the team’s hard efforts to address key underrepresented populations and also the trouble we have with these communities and how some of us can be ambassadors in our community for research.*

### Researcher evaluation: Post-session survey

Thirty-four researchers completed the initial post-session evaluation survey; respondents were investigators and other research staff (e.g., project manager, study coordinator) who played an active role in CFS planning and implementation. At least one post-session evaluation survey was completed for every project. Researchers were highly satisfied with the CFS process, rating the overall CFS service as Excellent [88% (*n* = 30)] or Good [9% (*n* = 3)]. Thirty-three (97%) researchers said they would recommend the CFS service to colleagues. Researchers rated the quality of specific parts of the CFS process highly as well, including communication with PaCER staff [88% Excellent (*n* = 30), 9% Good (*n* = 3)], session facilitation [91% Excellent (*n* = 31), 9% Good (*n* = 3)], and recruitment guidance, when applicable [78% Excellent (*n* = 18), 17% Good (*n* = 4)].

In open-ended responses, researchers further elaborated on positive aspects of the CFS, including comprehensive guidance and assistance from PaCER staff while preparing for the session, meeting structures that facilitated strong engagement from attendees and elicited useful and applicable feedback, skillful session facilitation, and high-quality key takeaway summary documents. Many researchers had no suggestions for improvement; recommendations received were related to session length and clarification of research team responsibilities in the CFS process. In rare instances when research teams experienced difficulties or delays in recruitment, researchers reported that receiving support, guidance, and assistance from PaCER helped alleviate these challenges and yielded successful recruitment. Common themes and illustrative excerpts from open-ended researcher initial evaluation survey responses are summarized in Table 5.

**Table 5.** Common themes from researcher post-session evaluation survey responses

Question	Response themes	Sample responses
What went well with the Community Feedback Session(s)?	Comprehensive guidance and assistance from PaCER staff while preparing for the session Meeting structure that facilitated strong engagement from partners Recruitment-related support and mentorship from PaCER staff, when applicable Elicitation of useful and applicable feedback Skillful session facilitation High quality key takeaway summary documents	*Great coordination and planning before the session. I felt supported through the process of creating recruiting information, gathering our cohort, and in making the material to be used during the session.* *The leadership from [PaCER staff], the facilitation was brilliant, the summary doc was very thorough and aesthetically pleasing. Their guidance was critical throughout. We had a very positive experience and I am so glad we did this.* *[PaCER staff] expertly led us through the creation of our presentation materials and facilitation guide, which helped our participants get a full understanding of our project and be able to provide excellent and well-informed feedback to our study team.* *The feedback sessions were incredibly helpful for learning about recruitment and retention strategies, understanding community-perceived burdens of the study, and where we can increase the value of science. We also learned strategies to promote trust and rapport between our research team and the larger community we aim to impact.*
How can future Community Feedback Session(s) be improved?	Offer options for bi-directional communication during sessions Provide further clarification of research team responsibilities in the CFS process Consider altering session length No suggestions for improvement	*I have very little feedback. It was excellent.* *I think everything went perfectly. We had challenges with recruitment but the [PaCER] team helped us in every way they could.* *I’d definitely recommend that the CFS team additionally put clear parameters on scope early in the process to help guide the client to a focused and purposeful goal.*

### Researcher evaluation: Follow-up survey

All researchers who have come due to complete their 6–12-month follow-up evaluation surveys (*n* = 16) have completed them. In their responses (summarized in Table 6), researchers noted specific changes they made to their projects as a result of feedback received during CFSs, including revising project timelines, amending study protocols, expanding study eligibility criteria, developing new plans and materials to facilitate communication with study participants, altering intervention content and delivery modalities, simplifying consent forms, and revising a variety of other study-related materials like logos, websites, flyers, infographics, videos, and recruitment scripts. Some researchers also described how they applied CFS feedback to other projects within their research portfolios, including refining the focus of future research projects and incorporating more generally applicable feedback about research priorities and recruitment considerations into subsequent grant applications. While most researchers noted that they did not face significant challenges incorporating feedback into their projects, some shared that financial and structural limitations (e.g., limited budget/personnel time, regulatory hurdles) made it difficult to apply some of the feedback received or noted that it was challenging to balance some areas of feedback when opinions differed within the group.

**Table 6.** Common themes from researcher 6-12-month follow-up evaluation survey responses

Question	Response themes	Sample responses
How, if at all, did your research project evolve or change due to the feedback you received in the session(s)?	Revised project timelines Amended study protocols Expanded eligibility criteria Developed new plans and materials to facilitate communication with study participants Altered intervention content and delivery modalities Simplified consent forms Revised study-specific materials (e.g., logos, websites, infographics, videos, recruitment scripts) Incorporated feedback about research priorities and recruitment considerations into future projects and grant proposals	*We heavily amended our approach to recruitment based on feedback we received. We updated recruitment guides and standard operating procedures. These changes were also reflected in our web-based database. The feedback helped us refine the processes and materials so that they were simpler and easier to use.* *We updated our recruitment materials to better match the advice we were given…We also met with our study physician and were able to loosen the medical criteria of the study.* *We modified recruitment scripts, processes, communication plans (intensity and type of communication), intervention delivery modalities, and developed a series of overview documents to provide more information to participants about what to expect in the study.* *Through these sessions we created and refined the application for the intervention arm of our trial. Without the sessions, we would have created the intervention without the benefit of end-user recommendations and feedback.* *This work was done in the pre-proposal phase and directly incorporated into the proposal planning and application itself.* *We have refined our focus for future projects, including recent grant applications, where we proposed to build on insights from the feedback sessions to develop and disseminate resources supporting equitable recruitment in lupus clinical trials.*
What challenges, if any, did you face in incorporating the feedback you received into your study?	Limited budget/personnel time Regulatory hurdles Difficulties balancing some areas of feedback when opinions differed amongst session participants Deciding how to prioritize feedback No challenges experienced	*Mostly that some of their suggestions would have been either too expensive or were simply impossible within the bounds of what REDCap was capable of.* *We were not able to amend all the study documents we wanted to amend due to regulatory and financial barriers.* *There were a few opinions that differed within group and so we were not able to accommodate every single voice.* *Some of the feedback gathered was very relevant but outside the scope of our work and control (e.g., insights for regulatory bodies).*
In what ways, if any, did the session(s) affect your team’s understanding of community engagement in research or ability to engage community, patient, or other partners in research?	Enhanced understanding of the patient/community experience Increased knowledge and understanding of engagement Contextualized issues of mistrust in research Built intention to incorporate engagement earlier in the research process and prior to launching future studies Demonstrated the value of engagement in building trust with patient/community partners	*The engagement sessions greatly improved our research team’s understanding of community engagement…Based on our experiences and appreciation of the CFS, we have modeled and incorporated components of the CFS approach in an ongoing, smaller-scale community engaged research project, as well as in proposals.* *Gave us a sense of the true value of seeking feedback from community and stakeholder partners early in the research process. That [PaCER staff] made it so smooth from our perspective and lowered barrier to entry made it so that we will now seek out this type of engagement in future projects.* *The sessions demonstrated clearly to me that well designed clinical research studies require careful collaborative pre-planning.* *I learned that we should incorporate the community earlier on in the process. We collected feedback on documents that our PIs considered final. The community should have been part of the design process.* *I was really impressed by how engaged participants were. Multiple participants offered to help with future video tools when I reached out to thank them after the session.* *The CFS was an important way for the UNC-based team to build relationships with the partners and earn their trust.*

Researchers also noted the ways in which conducting CFSs affected their understanding of engagement in research or ability to engage partners in the research process, describing enhanced understanding of the patient/community experience and intention to incorporate engagement earlier in the research process and prior to launching future studies. In general, researchers praised CFSs for providing an “excellent opportunity” to connect with partners and gain “invaluable feedback” to improve their research.

### Researcher evaluation: Long-term impact

The longer-term impacts of CFSs are still being assessed, as many were completed within the last 18–24 months. However, during periodic written and verbal check-ins with PaCER staff, some researchers have reported that CFSs resulted in notable outcomes such as successful acquisition of grant funding, development of conference presentations and manuscripts, sustained contact and further collaboration with CFS attendees, incorporation of study team-led CFSs and higher-level engagement approaches into future projects, and improvements in participant recruitment and retention.

## Discussion

Through the development, piloting, and evaluation of our CFS service, the PaCER Program has adapted the CE Studio model [17] into a scalable approach that requires minimal resources, facilitates capacity building within research teams to conduct future engagement without external support, and leverages virtual technologies. Evaluation data from researchers and partners support the acceptability, feasibility, and utility of this modified approach and indicate that the CFS service meets researcher needs, informs research in an impactful way, and provides value to partners. Existing publications have described implementing CE Studios within specific studies or projects [27–33], conducting CE Studios virtually [22,34–36], using CE Studios to engage different communities and populations [37–46], and developing “expanded” and “multisession” CE Studio approaches [47,48]; yet, there are few published examples [18] of implementing, reproducing, and/or adapting the CE Studio model to suit varied institutional contexts and capacities, a gap that this paper sought to address.

Throughout our adaptation process, we ensured that key components of the CFS approach reflect core principles of CE [1]. For example, our researcher-led recruitment process facilitates trust and strengthened partnerships; neutral facilitation of sessions and sharing of plain-language materials promotes bidirectional communication, culturally centered practice, and inclusivity; and partner compensation acknowledges the value of community knowledge and expertise. Our partner and researcher evaluation surveys also include items that map onto these principles, including questions about comfort sharing thoughts and ideas (trust, open communication), perceived contributions made to research projects (perceived influence, value), effects of CFS participation on understanding of research (expanded knowledge, capacity building), perceived benefits of CFSs (value), changes made to research projects based on feedback received from partners (improved projects/programs), effects of CFS involvement on understanding of CE and ability to engage partners in research (expanded knowledge, capacity building), continued contact/collaboration with CFS attendees (strengthened partnerships), and outcomes related to dissemination and future funding (sustainability).

Evaluation responses from researchers and CFS attendees indicate that our modifications to the CE Studio – specifically, altering processes related to session recruitment, partner orientation, and session planning and delivery – have been acceptable to researchers and partners and have positively impacted their experiences with and involvement in the CFS service. As reported by researchers and partners, researcher-led recruitment resulted in discussion groups that provided comprehensive, relevant, and appropriate feedback and included a range of perspectives, opinions, and experiences. Our positive experiences implementing virtual CFSs align with what others have described in publications when implementing project- or study-specific virtual CE Studios [22,34–36]. Additionally, partners’ suggestions related to session preparation did not include mention of or desire for additional onboarding or virtual touchpoints with the research team or PaCER staff, though this is not something we asked about explicitly in our evaluation process. We plan to incorporate further evaluation of partners’ perceived level of preparedness to participate in a CFS given abbreviated onboarding – which we did not formally assess in post-session surveys – as a future direction of our evaluation process.

Feedback received from researchers provides compelling evidence that CFSs can both help build research team capacity for conducting future sessions without PaCER support and for implementing other engagement approaches in their work. Active involvement in the CFS process seems to provide researchers with insight into how engagement works and elucidates processes related to the planning, implementation, execution, and evaluation of engagement activities. The CFS model allows researchers to build engagement knowledge and capacity in a supportive environment through ongoing consultation, communication, and support from PaCER staff. In informal communications with our team, some researchers expressed the realization that they had more connections to potential partners than they initially thought and were able to leverage existing connections with their own networks of patients, community members, and clinical partners during CFS recruitment. Although long-term impacts of CFSs implemented to date are still being assessed, researchers have reported independently implementing additional CFSs in their projects (e.g., with other groups of interest-holders, at later stages in the research process), incorporating CFSs and other engagement approaches – such as advisory boards and working with partners as co-investigators – into future studies and proposals, using feedback from CFSs to inform grant applications and other research projects within their portfolios, and sustaining contact and further collaboration with CFS attendees, which was previously noted as an unintended positive outcome of some CE Studios [17,18].

While we developed researcher-focused evaluation processes to assess CFS-related outcomes beyond the initial survey, we have not conducted follow-up surveys with partners. Thus, we cannot report longer-term impacts of CFS participation on partners’ perceptions and understanding of research, or involvement in future research or engagement activities, though this topic has been discussed in other publications in the context of CE Studio participation [49]. Piloting partner follow-up surveys to assess whether CFS participation influenced future engagement, trust, or self-efficacy in research contexts could help explore this further. Furthermore, given the CFS approach’s reliance on researcher-led screening and recruitment, PaCER does not collect demographics of CFS attendees and thus we are unable to compare survey respondent versus total attendee composition through a nonresponse analysis. Additionally, we did not assess researchers’ engagement experience before their CFSs, so we do not know if researchers with more engagement experience would rate their CFS experience differently than those with less experience. Due to staffing limitations, we currently conduct all CFSs in English. While there has been some work describing utilization of the CE Studio model for multilingual populations [48,50], further exploration is needed to determine the feasibility, acceptability, and utility of CFSs conducted in languages other than English. Lastly, we cannot speak to the comparative effectiveness of the CFS process versus the CE Studio model, as doing so would require formalized testing of the different approaches within a controlled environment, which is beyond the scope of this paper and our work to date. However, comparing the effectiveness of various CE Studio model adaptations could be the topic of a future research study and would help build the evidence base related to research engagement.

The CFSs conducted through PaCER are driven by researcher-initiated requests. Prior to 2025, the number of sessions implemented rose steadily year over year as we scaled up the service (10 sessions in 2022, 13 in 2023, 16 in 2024); notably, we did not experience an increase in sessions in 2025 (8 sessions have been delivered to date in 2025) likely due to an increase in service charge rates and turmoil in the federal funding landscape. Most researchers utilizing the CFS service have funded their projects through departmental funds, existing grant funds, or budgeting for CFSs in grant proposals. To further bolster early-stage engagement (i.e., engagement during proposal development and study planning), PaCER has instituted an Engagement Voucher Program through which researchers can receive funding to support pre-award engagement activities like CFSs [20,23]. Our focus on reducing the number of PaCER staff hours required to implement a CFS has positioned us to not only meet increasing demand and grow the service, but also allows the service to remain affordable for research teams with limited budgets. Notably, we have achieved attendee counts and turnaround times from initial service request to session delivery comparable to published CE Studio metrics [17,18], despite our reduced staffing structure; this ability to maintain service efficiency with limited resources further highlights the scalability and affordability of the CFS approach.

Developing institutional infrastructure and service offerings to support engagement in research – like many CTSAs have done – is critical to promoting researchers’ utilization of and capacity to implement evidence-based engagement methods. We successfully modified the CE Studio model to promote scalability within our institution, showing promising short- and intermediate-term outcomes. In addition to continuing to implement CFSs and enhance the quality of the service within our institution, we believe this model may provide value to investigators and research staff across the CTSA consortium and beyond. To further inform and strengthen the translational value of the CFS approach, others could consider using an implementation science framework and CE implementation principles to guide their adoption or uptake of the CFS model [1]. Additional future directions include further evaluation of long-term impacts of CFSs on research projects, more robust evaluation of CFS attendee experiences (e.g., perceived preparedness for sessions, longer-term impacts of session participation), conducting CFSs in languages other than English, and further study of the CFS approach in comparison to other engagement methods.

## Supporting information

10.1017/cts.2026.10745.sm001 Frank et al. supplementary material 1 Frank et al. supplementary material

10.1017/cts.2026.10745.sm002 Frank et al. supplementary material 2 Frank et al. supplementary material

10.1017/cts.2026.10745.sm003 Frank et al. supplementary material 3 Frank et al. supplementary material

10.1017/cts.2026.10745.sm004 Frank et al. supplementary material 4 Frank et al. supplementary material

10.1017/cts.2026.10745.sm005 Frank et al. supplementary material 5 Frank et al. supplementary material

10.1017/cts.2026.10745.sm006 Frank et al. supplementary material 6 Frank et al. supplementary material

10.1017/cts.2026.10745.sm007 Frank et al. supplementary material 7 Frank et al. supplementary material

10.1017/cts.2026.10745.sm008 Frank et al. supplementary material 8 Frank et al. supplementary material

## Supplementary material

The supplementary material for this article can be found at https://doi.org/10.1017/cts.2026.10745.

## Figure 1. Long description

The flowchart outlines the steps involved in requesting and implementing a Community Feedback Session (CFS) with the Patient and Community Engagement in Research (PaCER) Program. The process begins with PaCER receiving a request for a CFS from the research team. This is followed by a consultation phase where orientation to the CFS process, sharing planning resources, defining the focus or topic of the CFS, and formulating discussion questions occur. The research team recruits partners for the CFS, creates a plain-language project presentation, and coordinates with PaCER and partners to schedule the CFS. PaCER prepares a facilitation guide with discussion prompts, develops an information sheet, and supports the research team with recruitment and presentation preparation. Partners confirm CFS attendance and review the information sheet. The Community Feedback Session itself is a 1.5 to 2-hour virtual meeting where the researcher delivers a presentation, the PaCER facilitator leads the discussion, and partners provide feedback. After the session, the research team compensates partners and completes an evaluation. PaCER prepares a summary report of key takeaways and sends evaluation surveys to researchers and partners.

Navigate back to Figure 1..

## Figure 2. Long description

The table compares research and engagement activities, focusing on key differences. It has two columns labeled ‘Research’ and ‘Engagement,’ each with several rows detailing specific aspects. The ‘Research’ column notes that the goal is to collect data and produce generalizable knowledge, participants are studied, informed consent is required, individuals receive incentives, data is gathered through methods such as surveys and interviews, and IRB approval is necessary. The ‘Engagement’ column states the goal is to work collaboratively to enhance specific research projects, people are partners, no informed consent is needed, individuals are compensated for their time and expertise, input is gathered through methods such as feedback sessions, and engagement is not considered research but may seek a NHSR IRB determination.

Navigate back to Figure 2..

## Figure 3. Long description

A table comparing Community Feedback Sessions, Community Advisory Boards, and Research Focus Groups. The table has three columns and three rows. The first column describes the Community Feedback Session, which involves gathering one-time feedback to inform specific parts of a research project, bi-directional interaction between researchers and community members, ad hoc convening of community partners, and is suitable for shorter timelines and limited budgets. The second column outlines a Community Advisory Board method, which provides ongoing guidance and oversight for a research project or organization, involves bi-directional agendas, discussions, and progress reporting, consists of a consistent group of community partners, and requires more resources for mid- to long-term engagement. The third column details the Research Focus Group, which focuses on qualitative data collection, follows a uni-directional approach where researchers guide and participants answer questions, is approved or deemed exempt by the Institutional Review Board, involves consented participants, and aims to gather and analyze data to produce generalizable knowledge.

Navigate back to Figure 3..
