# Supplementary material for: Community feedback sessions: An adaptation of the community engagement studio model to enhance scalability
Source: J Clin Transl Sci. 2026 May 6;10(1):e91. doi: 10.1017/cts.2026.10745 (PMC13237187; doi:10.1017/cts.2026.10745)
Supplement: Frank et al. supplementary material 1 — Frank et al. supplementary material [file S2059866126107456sup001.pdf]

Enhance your research with community feedback

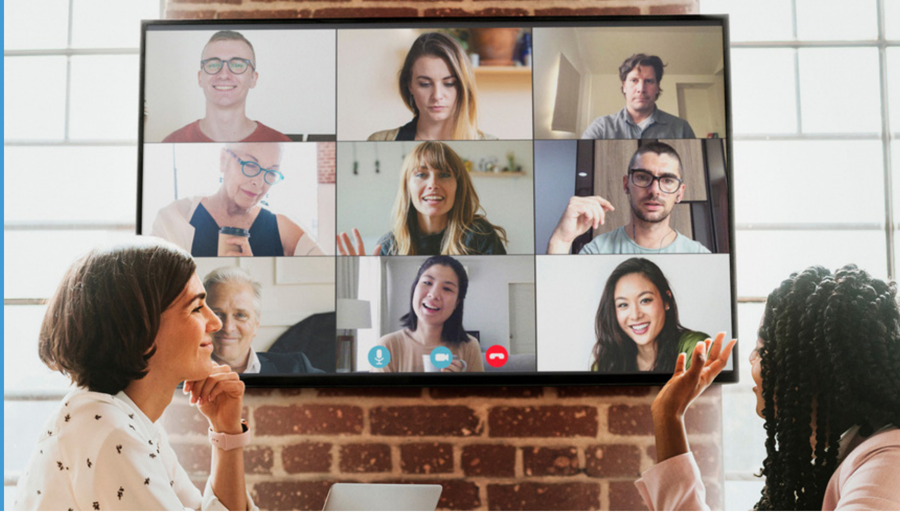

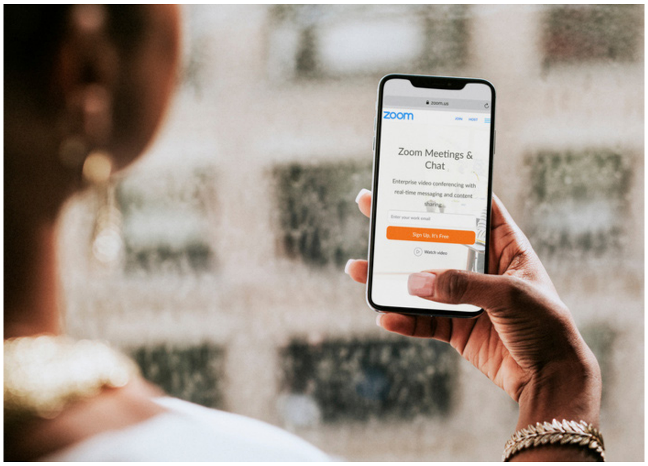

WHAT IS A COMMUNITY FEEDBACK SESSION (CFS)?

Our trained staff are available to facilitate Community Feedback Sessions for researchers interested in obtaining patient and community partner input on the design, implementation, or dissemination of a research project. These sessions last 1.5-2 hours and aim to elicit authentic and constructive feedback from a small group of partners.

ABOUT US

The **Patient and Community Engagement in Research (PaCER)** program at NC TraCS works with researchers to improve the health of all North Carolinians by promoting the inclusion, engagement, and recruitment of diverse communities and patients.

If you are interested in community feedback for your project, request a consultation at **tracs.unc.edu** to learn more and inquire about availability.

HOW CAN A CFS HELP MY RESEARCH?

- Partner insight and perspectives can inform research questions and approaches, leading to studies that are more relevant and less burdensome to potential participants
- Partners can help to refine proposals and identify potential barriers and challenges to recruitment and retention
- Partners can provide input on the cultural and linguistic acceptability of recruitment materials, consent forms, or data collections tools like surveys or interview guides

Community Feedback Session FAQ

HOW IS A CFS DIFFERENT FROM A RESEARCH FOCUS GROUP?

- A CFS is not considered research. It is not intended to collect data or produce generalizable evidence. It *does* solicit partner guidance to inform research at any stage of the project
- A research focus group is designed to collect data. Research focus groups must be approved (or determined exempt) by an Institutional Review Board (IRB). Focus group participants typically are required to complete an informed consent process

HOW MUCH WILL IT COST?

- **Our Rates:** For a current list of NC TraCS rates, visit **tracs.unc.edu/rates**.
- **MOU:** A Memorandum of Understanding with our team is required before we begin work on your project
- **Estimated PaCER time:** 8 hours per session with an additional 8 hours of overall prep time
- **Additional costs:** We recommend compensating each attendee $100 per session

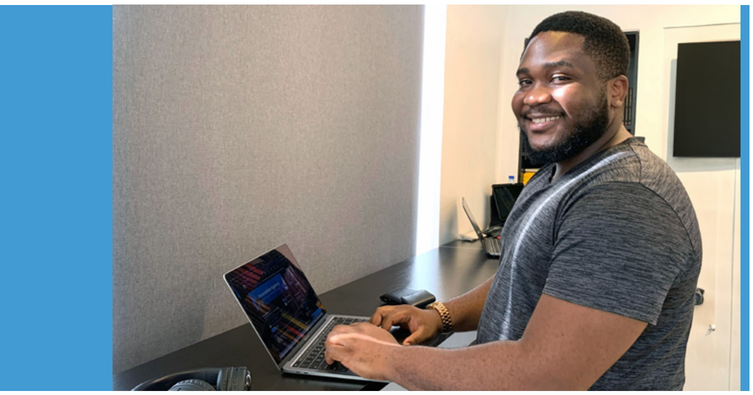

WHAT HAPPENS DURING A CFS?

- A 5-10 minute presentation by the researcher about the project
- 2-4 questions posed to a group of partners/attendees
- Discussion guided by a PaCER facilitator that elicits authentic and constructive feedback

WHAT CAN YOU EXPECT FROM PaCER?

- Advice on community-friendly materials for your session (flyers, information sheets, the session presentation)
- Guidance on recruiting attendees
- Helpful templates and resources
- Development of the facilitator's guide
- A facilitator and note-taker for your session
- A feedback report

WHAT IS EXPECTED OF YOUR PROJECT TEAM?

- Meet with PaCER to develop the goals and timeline for your session
- Recruit 4-8 individuals from a community or population of interest
- Prepare a plain language 5-10 minute presentation about your project
- Attend the CFS
- Teams on average commit 10-15 hours of staff time
- Provide compensation to attendees
- Complete an evaluation of the CFS

To find out more or get started with a CFS:

REQUEST A CONSULTATION
