# Supplementary material for: Community feedback sessions: An adaptation of the community engagement studio model to enhance scalability
Source: J Clin Transl Sci. 2026 May 6;10(1):e91. doi: 10.1017/cts.2026.10745 (PMC13237187; doi:10.1017/cts.2026.10745)
Supplement: Frank et al. supplementary material 2 — Frank et al. supplementary material [file S2059866126107456sup002.pdf]

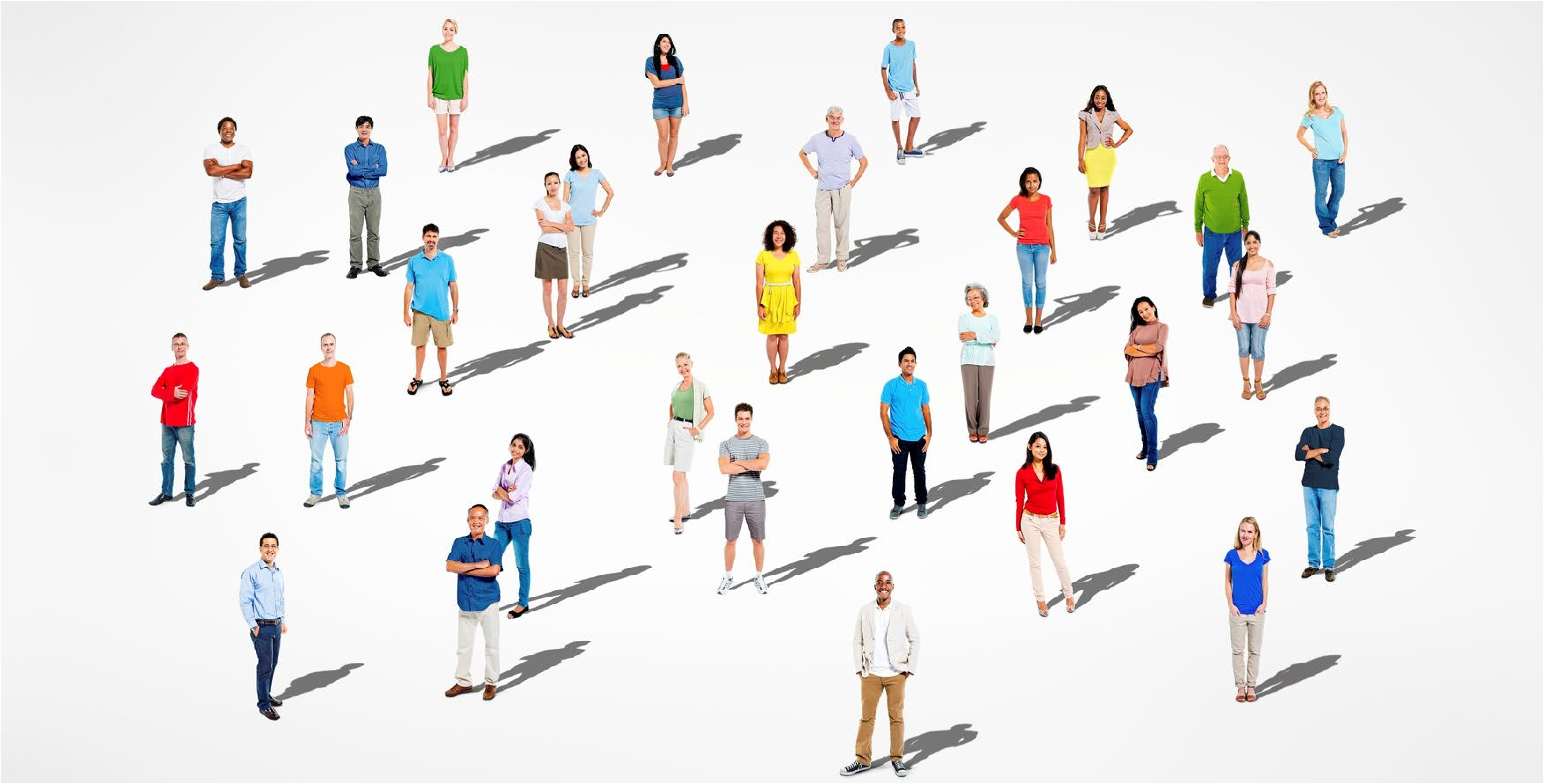

Community Feedback Session

A Service Offered by the

Patient and Community Engagement in Research (PaCER) Program

UNC
SCHOOL OF MEDICINE
North Carolina Translational and Clinical Sciences Institute

What is a community feedback session (CFS)?

A Community Feedback Session is:

- A brief (1.5 or 2 hour) consultative session for researchers interested in obtaining input from patients, caregivers, health care providers, community members and other partners on the development, implementation or dissemination of a research project.

It's not:

- A standing advisory board, a tool for recruiting research participants, or a data collection or research methodology.

How can it help your project?

- Create studies that are more relevant and less burdensome to participants.
- Refine proposals and identify challenges to recruitment and retention.
- Assess feasibility and appropriateness of the project for a population or community.
- Develop understandable and culturally appropriate research materials.

What's the difference?

Community Feedback Session

One-time feedback to inform specific part(s) of a research project

Bi-directional: researcher presents & community members provide input

Community partners are convened ad hoc; share feedback & lived experience

Engagement option for a shorter timeline & limited budget

Community Advisory Board

Ongoing guidance/oversight for a research project or organization

Bi-directional: agendas, discussions, progress reporting, etc.

Consistent group of community partners provide oversight & advice

Mid- to long-term engagement option; requires more resources

Research Focus Group

Qualitative data collection

Uni-directional: researcher follows guide & participants answer questions

Approved, or deemed exempt, by IRB

Participants consented to participate

Goal is to gather and analyze data, produce generalizable knowledge

A community feedback session consists of:

1

A brief (5-10-minute) presentation by a researcher that describes their project and purpose of the feedback session

2

A set of specific discussion prompts posed to the group of community partners

3

Discussion guided by a neutral facilitator to elicit authentic and constructive feedback

What can you expect from us?

- Advice on creating community-friendly materials for your session
- Guidance on recruiting attendees
- Helpful templates and resources
- Development of the discussion prompts, facilitation guide, and 1-page information sheet
- A facilitator and notetaker for your session
- A summary of key takeaways from each session
- A referral to the Recruitment & Retention team (if helpful)

What do we expect from your team?

- Recruit 4-8 individuals from a community/population of interest for your feedback session
- Create & send a calendar invitation to feedback session participants (our team will provide a Zoom link)
- Prepare a community-friendly 5-10-minute presentation and attend the CFS
- Provide compensation to attendees *(typically, research teams choose to compensate CFS participants via gift card; this requires submitting a Not Human Subjects Research application to the IRB so that your engagement activities have an IRB#)*
- Share a link to our CFS participant evaluation survey in any follow-up communications with CFS participants
- Complete a short evaluation survey after the session and then another 6-12 months later to assess impact

Need help with recruitment?

We can assist by:

- Providing example participant screening surveys that have been used in prior projects
- Reviewing your recruitment flyer and/or email
- Emailing our network of community partners about your CFS (for projects focused on the general population)
- Referring you to the Recruitment & Retention Program for specific guidance

Need help with the IRB*?

We can assist by:

- Providing an example Not Human Subjects Research (NHSR) IRB application
- Providing example language to include in your NHSR IRB application
- Sharing resources and links to learn more about the NHSR IRB determination

*****We recommend submitting a NHSR IRB application as the <u>first step</u> in preparing for your CFS (prior to starting recruitment for your session)

Tips for CFS recruitment

Research teams who have contracted with the NC TraCS CFS service have found this general process to be helpful when conducting feedback session recruitment:

1. Identify 3-4 potential CFS dates/times that will work for both your team & the NC TraCS team. NC TraCS staff will place holds on their calendars.
2. Develop an online screener (e.g., Qualtrics, REDCap) to assess eligibility & availability of potential CFS participants. Include the 3-4 potential CFS dates/times you identified as options in the screener.
3. Develop plain language for a brief recruitment email and/or flyer & include a link or QR code to the online screener – share the recruitment materials with your networks & within the communities you wish to engage in your feedback session.
4. Review online screener responses & identify a date/time where 4-8 people are available to attend a session. *As you review screener responses, consider how you will be including a diverse group of people & perspectives in the conversation.*
5. Email attendees to formally invite them to the feedback session. Share the final date/time of the session, confirm their attendance, & let them know you will be sending a calendar invitation shortly. *It can be helpful to have 1-2 back-up participants in case there are last minute dropouts or no-shows.*
6. Once the date/time is finalized, let the NC TraCS team know so we can release calendar holds & create a Zoom link for your session. *Our team prefers to create Zoom links so we can manage polls & the session recording.*
7. Create a calendar invitation that includes the Zoom link from NC TraCS – send to all confirmed attendees & NC TraCS staff.
8. Send a reminder email to all confirmed attendees 2-5 days before the session. Include the 1-Page Information Sheet (prepared by NC TraCS) in your reminder email & attach it to the calendar invitation. Ask participants to briefly review the Information Sheet, which will contain additional information about the session.
9. Send a final reminder email the day of the feedback session.

How much work is a CFS?

It varies! We estimate your project team might spend:

- 4-6 hours on planning/preparation for the session, including attending meetings, reviewing materials, and creating a plain language brief presentation
- 2-5 hours recruiting attendees
- 1.5-2 hours attending each CFS
- 1-2 hours processing compensation

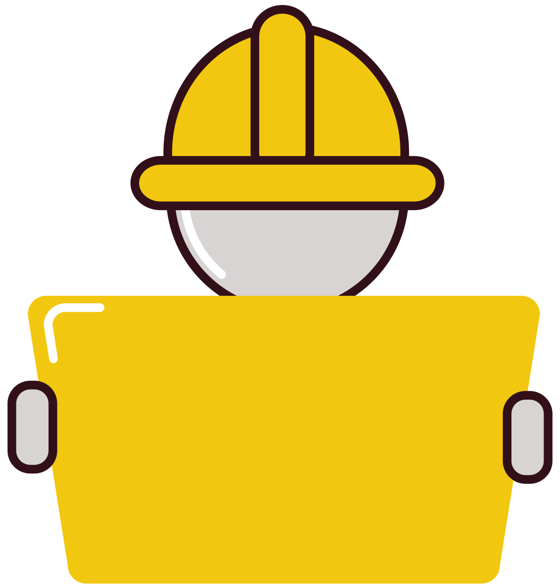

Preparing for the CFS

PaCER

Study Team

Kick-off meeting

CFS Worksheet

Your team fills out the worksheet and returns it to PaCER. We may reach back out with questions and suggested edits. PaCER will use the worksheet responses to draft a Scope of Work (SOW) and develop the materials for your session.

SOW

Your team and PaCER sign a Scope of Work prior to the start of work.

NHSR IRB application

Your team prepares and submits a NHSR IRB application. PaCER can provide guidance & resources to support you through this process.

Start recruitment

Your team starts recruiting attendees for your session(s), based on the dates/times you identified in collaboration with PaCER.

Conducting the CFS*

PaCER

Study Team

Facilitation guide

PaCER develops the discussion prompts and facilitation guide for the CFS. This will be sent to your team for review and approval.

Project presentation

Your team creates a brief (typically 5 to 10 minute) plain language presentation that describes your project and purpose of the feedback session. PaCER can review and provide feedback on your presentation.

Information Sheet

PaCER prepares a 1-Page Information Sheet & Zoom link that you can send to your attendees in advance of the session.

Feedback session

The PaCER facilitator introduces the session. Your team presents the project. The PaCER notetaker records the meeting and monitors the chat. The PaCER facilitator engages the attendees in discussion. During the last 5 minutes of the CFS, attendees will be asked to complete a brief evaluation survey.

*Your team can expect to have 1-3 check-in meetings with PaCER prior to the CFS to review materials (e.g., guide, presentation, etc.) and discuss any other needs in advance of the session.

After the CFS

PaCER

Study Team

Compensation

Your team sends a "thank you" message and compensation to attendees.

Evaluation

Your team sends a link to our brief evaluation survey for CFS participants in any follow-up communications you have with attendees. Your team will receive a brief survey via email too, after all CFSs for your project have been completed.

Feedback summary

PaCER will prepare a summary of key takeaways gathered from the session. Consider sharing the summary with your session attendees.

Your team may want to consult with the Recruitment & Retention Team for implementation advice, if applicable.

Follow-up survey

6-12 months after your session, your team will receive another brief survey so PaCER can learn how the feedback sessions impacted your project.

Next steps: CFS Worksheet & Scope of Work

We will send you a CFS Worksheet to fill out with your team. The information you provide will document your project's needs and will ask for details including:

- Your project timeline
- Who your feedback session participants will be
- How you plan to recruit attendees for the CFS
- What information you'd like to take away from the session
- How you plan to use the feedback

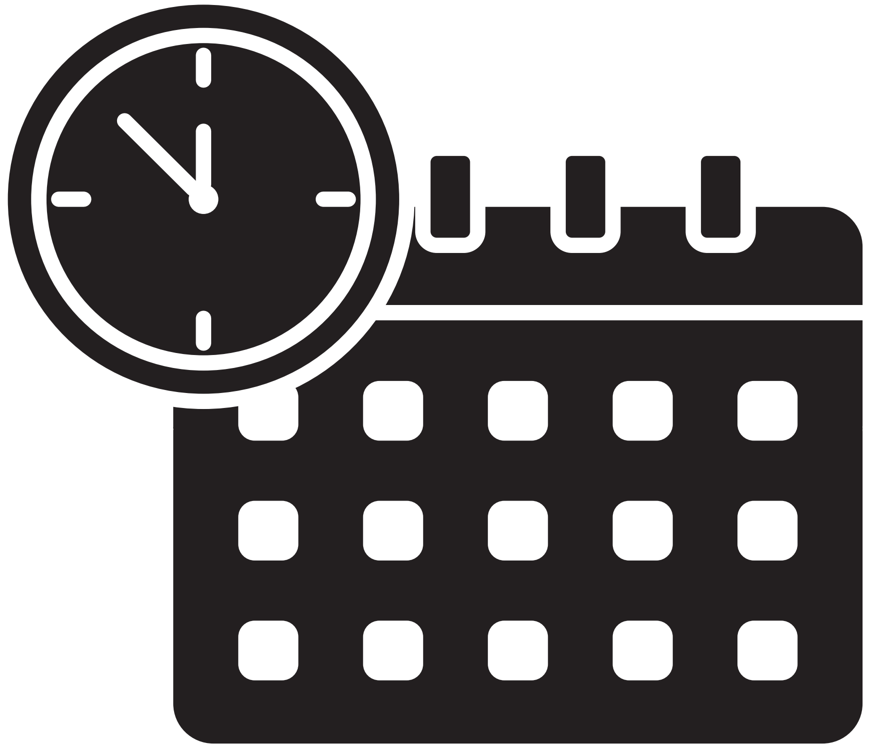

Approved worksheets are used to:

- Draft a Scope of Work
- Develop the discussion prompts for the session
- Inform an outline for your brief CFS presentation

We will also send you:

- A Scope of Work between PaCER and your team to indicate when work on the CFS will start and when billing begins. This must be signed before work continues.

Contracting with PaCER

Effective May 1, 2025, billing rates are as follows:

- Business hours (M-F, 8:00am-5:00pm ET): $113/hour (School of Medicine), $143/hour (non-School of Medicine), $151/hour (other schools within the UNC system), or $235/hour (non-UNC system schools). Rate categories will be determined by the project PI's primary department.
- All requests for services or support outside of business hours will be subject to an additional administrative charge of 1 hour per occurrence and per individual involved in the request. This administrative time is required to account for the coordination, documentation, and management of after-hours work. The 1-hour administrative charge will be added to the total service time for each individual working the request and will be billed in accordance with our standard billing practices.

**After-hours work is provided on a limited basis; it should be scheduled well in advance and must be confirmed or cancelled at least five business days prior to the event.*

Estimated Hours for 1 Community Feedback Session:

- **16 hours of PaCER staff time:**
  - 8 hours for preparation (ongoing consultation, guidance, check-in meetings, and material development)
  - 4 hours conducting the CFS (2 hours of facilitator time + 2 hours of notetaker time)
  - 4 hours for development of the key takeaway summary

Estimated Budget for 1 Community Feedback Session:

- **$2,608 - $3,088**
  - $1,808 (SOM), $2,288 (Non-SOM) for PaCER staff time *(These funds would be billed to your team by PaCER)*
  - $800 for CFS participant compensation *(Estimates reflect our recommended rate of $100/session/participant & recommended number of up to 8 participants per session. These funds are paid directly by your team to the feedback session participants; NC TraCS does not facilitate payment and final decisions about pay rates are determined by the study team)*

I want to conduct multiple feedback sessions…how will this affect the budget?

CFS Participant Compensation:

- You will need to budget for all participants across all sessions (e.g., 3 feedback sessions x 8 participants each x $100 = $2,400).

PaCER Staff Time:

- If each CFS will cover a different topic, 16 hours of PaCER staff time will be required for each session (e.g., 1 CFS to gather feedback about recruitment materials + 1 CFS to inform dissemination strategies = 32 hours of PaCER staff time). Distinct prompts, guides, and materials will need to be prepared for each session.
- If CFSs will cover the same topic, 8 hours of PaCER staff time will be required for each additional session (e.g., 2 CFSs to inform proposal development = 24 hours of PaCER staff time).

Additional Information

- PaCER typically does not have the capacity to conduct more than 3 CFSs per project (most teams conduct 1-2 per project).
- Since CFSs are an engagement activity meant to inform your research project, multiple CFSs can be most helpful if you'd like to hear from different groups of people or gather feedback at different stages of your project (you are not aiming to reach "saturation" as you would in qualitative research or produce "generalizable knowledge").

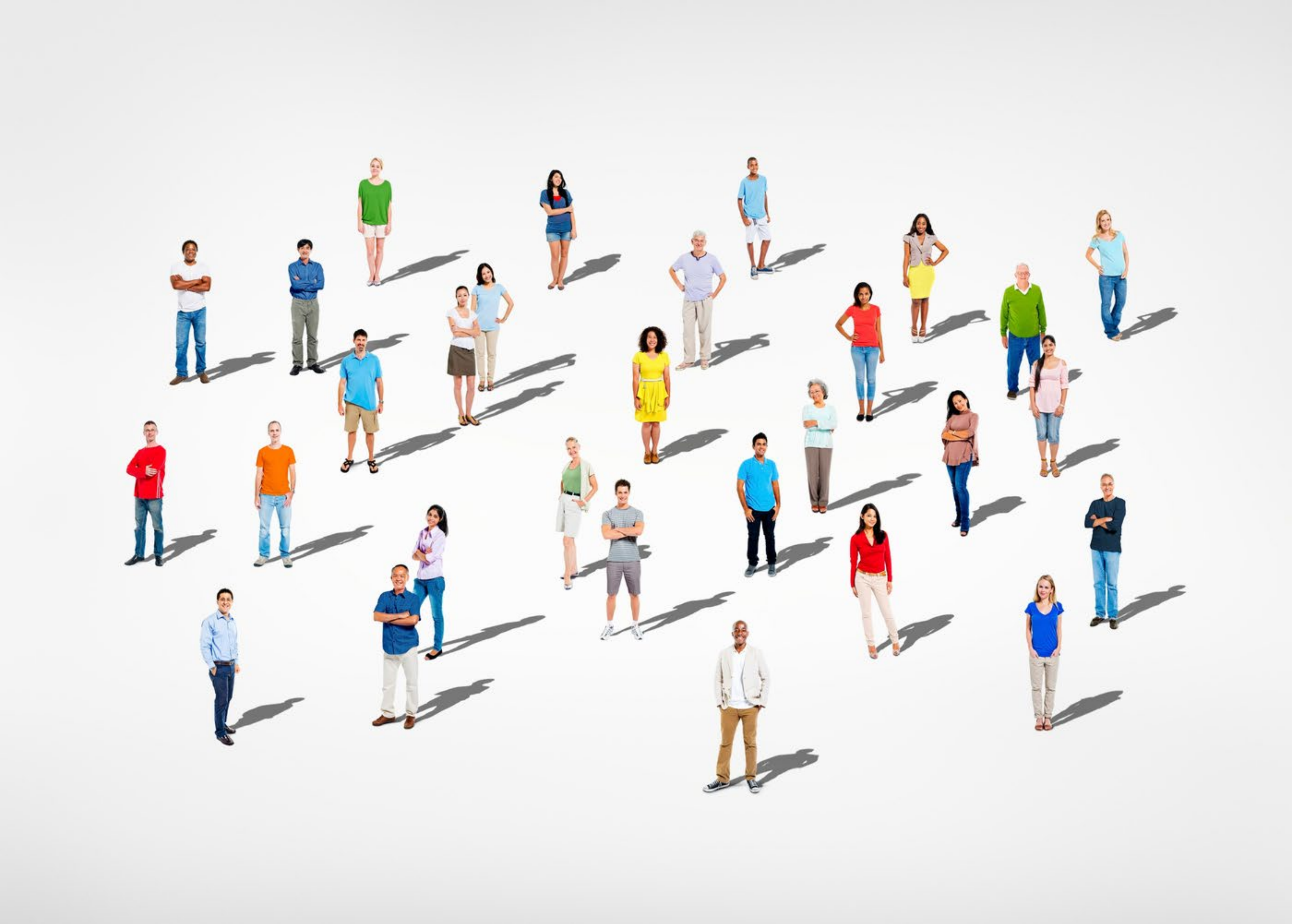

Image by rawpixel.com

Questions?

- Simone_Frank@med.unc.edu
- Ese_Aikhuele@med.unc.edu
- Nisha_Datta@med.unc.edu
- MaryBeth_Grewe@med.unc.edu
- Alicia_Bilheimer@med.unc.edu

Key References & Resources

Publications & Presentations about the PaCER Community Feedback Session service:

Datta N, Frank SC, Grewe ME, Yang C, Lawal K, Bilheimer A. Enhancing the Impact of a Community Feedback Session Service Through Ongoing Evaluation. Presented as a poster at the Association for Clinical and Translational Science's Translational Science 2024, Las Vegas, NV, April 3, 2024.

Seminal Publications about the CFS Model:

Joosten, Y. A., Israel, T. L., Williams, N. A., Boone, L. R., Schlundt, D. G., Mouton, C. P., Dittus, R. S., Bernard, G. R., & Wilkins, C. H. (2015). Community Engagement Studios: A Structured Approach to Obtaining Meaningful Input From Stakeholders to Inform Research. Academic medicine : journal of the Association of American Medical Colleges, 90(12), 1646–1650. https://doi.org/10.1097/ACM.0000000000000794

Kas-Osoka, C. N., Israel, T., & Ahonkai, A. (2022). 130 Examining the Role of Community Engagement Studios & Community Experts in Translational Research: A Thematic Analysis. Journal of Clinical and Translational Science, 6(Suppl 1), 8. https://doi.org/10.1017/cts.2022.43

Meharry-Vanderbilt Community Engaged Research Core, Vanderbilt Institute for Clinical and Translational Research. Community Engagement Studio Toolkit 2.0. https://www.meharry-vanderbilt.org/sites/vumc.org.meharry-vanderbilt/files/public_files/CESToolkit%202.0.pdf
