# Supplementary material for: Community feedback sessions: An adaptation of the community engagement studio model to enhance scalability
Source: J Clin Transl Sci. 2026 May 6;10(1):e91. doi: 10.1017/cts.2026.10745 (PMC13237187; doi:10.1017/cts.2026.10745)
Supplement: Frank et al. supplementary material 3 — Frank et al. supplementary material [file S2059866126107456sup003.pdf]

Community Feedback Session (CFS) Worksheet

We have created this worksheet as a resource for both your project team and the Patient and Community Engagement in Research (PaCER) team. It will document your needs and goals, and provide us with the information needed to prepare discussion questions for your community feedback session(s). The discussion questions are the prompts that the facilitator will pose to your attendees to elicit the kind of feedback you've identified as important to your project's success. Once your team has completed this worksheet, we will review it and ask any clarifying questions that might help us further refine the discussion questions. Work will begin work for your session when the worksheet is finalized and approved by our team, and a signed Scope of Work (SOW) is in place.

If you have any questions, need assistance filling out the worksheet, or would like to submit a completed worksheet to the PaCER team, please email Simone_Frank@med.unc.edu.

Expectations of your project team

Please review the expectations listed below and indicate your agreement by checking the boxes. If you have any questions or concerns, let us know before completing the worksheet.

☐ The project team has the time and capacity for this activity (we estimate 2-4 hours for preparation, 2-4 hours of recruitment, 1.5-2 hours attending each CFS, and 2 hours for arranging compensation).

☐ The project team will prepare and submit a Not Human Subjects Research (NHSR) IRB application for the CFS activities (this will allow your team to have an IRB# that you may use when acquiring gift cards for CFS participants).

☐ The project team will recruit 4-8 individuals from the community or population of interest to participate in each feedback session.

☐ Community feedback sessions are conducted via Zoom unless it poses a problem for the attendees. The CFS will be recorded for notetaking purposes.

☐ 1-2 project team members will attend each CFS to present a plain language explanation of the project and purpose of the CFS, and answer any clarifying questions from attendees.

☐ The project team will compensate attendees. PaCER recommends a rate of $100 per attendee per session.

☐ The project team will complete an evaluation survey immediately after the session(s) and a follow-up survey after 6-12 months.

Your Research Project

In the table below, please tell us about the research project that the community feedback session(s) will provide input on.

	Please write your responses in the cells below:
Project title	
IRB number	
Principal Investigator name & email	
Main point of contact for the project team & email	
Funding type	
Project overview (summary of the problem or research questions your project aims to address)	
Stage of research	
Target population for the study/project	
List the interest-holders who might be impacted by the research project	
Anything else you'd like us to know about your project?	

Community Feedback Session Details

The information below will help us begin planning for your community feedback session(s).

	Please write your responses in the cells below:
When do you need to collect this feedback by? Is your timeline fixed or flexible?	
Please propose several date/time options for your feedback session(s).	
Who would be your ideal session attendees?	*Aspects to consider for this question: demographics or backgrounds; experience with a certain health condition; roles (e.g., patients, caregivers, community members, clinicians, etc.)*

How will you go about finding and recruiting attendees?	
How many feedback sessions do you envision?	*If you are considering more than one session, please describe how you plan to divide up attendees across sessions (e.g., by demographics, by roles)? We can help you think through power and group dynamics if this is a consideration.*
What information would you like to walk away with from the feedback sessions?	*Things to consider: Are there specific parts of your study for which you'd like to gather feedback? What areas of your project do you hope to impact with community feedback? Are there parts of your project that cannot be changed (because of budget, already approved protocol, etc.)?*
How do you plan to use the feedback that is gathered?	
Anything else you'd like to tell us about?	
