# Supplementary material for: Community feedback sessions: An adaptation of the community engagement studio model to enhance scalability
Source: J Clin Transl Sci. 2026 May 6;10(1):e91. doi: 10.1017/cts.2026.10745 (PMC13237187; doi:10.1017/cts.2026.10745)
Supplement: Frank et al. supplementary material 4 — Frank et al. supplementary material [file S2059866126107456sup004.pdf]

UNC
SCHOOL OF MEDICINE
North Carolina Translational and Clinical Sciences Institute

Patient and Community Engagement in Research (PaCER) Program

Community Engagement Activities as NHSR

Guidance for distinguishing between research and community/patient engagement, and for understanding IRB processes related to engagement activities as Not Human Subjects Research (NHSR).

Disclaimer
Although this is a quick guide for navigating IRB submissions related to community/patient engagement activities, the suggestions and guidance offered here are subject to change and are contingent on university operating procedures. Please refer to UNC's IRB and Office of Human Research Ethics (OHRE) website for the most up-to-date guidance.

Is My Activity "Research" or "Engagement"?

While research and engagement activities might look similar, their purposes and intended outcomes typically differ. Below are some of the key differences to consider. For further guidance and examples, contact the NC TraCS PaCER team.

Please note: the term "partner" is used throughout this guide to refer to the people and organizations invested in a research area or project and/or affected by its outcomes.

Research	Engagement
The goal is to **collect data** and **produce generalizable knowledge**	The goal is to **work collaboratively** with partners to **enhance the quality of a specific research project**
**Participants** are "studied"	People are **partners** in the research process
Research participants complete the **informed consent** process	Generally, **no informed consent** process is needed
Individuals receive **incentives for participation**	Individuals are **compensated for their time & expertise,** similar to consultants
Participant input or data is often gathered through **surveys**, **focus groups**, **interviews**, etc.	Partner input is often gathered through **feedback sessions**, **advisory board membership**, etc.
**The project must be approved or deemed exempt** by the Institutional Review Board (IRB)	**Engagement is not considered research**, but research teams may pursue a "Not Human Subjects Research" determination from the IRB

If your activities are considered engagement, then applying for an NHSR determination is appropriate. Otherwise, we'd recommend submitting your application under a different category—IRBIS's online submission FAQ can walk you through the process.

Not Human Subjects Research IRB Applications: Basic Considerations

What is "Not Human Subjects Research" (NHSR)?

According to UNC's SOP 501, NHSR includes any activities that do not involve "research", "human subjects", or a "clinical trial" (as defined in SOP 6001), and thus does not require IRB oversight. As such, most engagement activities are classified as NHSR.

Ultimately, it is the IRB's responsibility to determine if a project is Not Human Subjects Research (NHSR) or Human Subjects Research (HSR). However, it is the Principal Investigator's responsibility to initially decide whether a project should be submitted to the IRB as NHSR or HSR.

Why is it helpful to submit engagement activities as NHSR to the IRB?

Although teams are not required to submit NHSR activities for IRB review, it is recommended for the following reasons:

- It allows the IRB to ultimately determine whether or not activities constitute NHSR vs. HSR.
- It provides teams with IRB documentation that some scholarly journals may require in order to publish on any engagement activities.
- It provides teams with an IRB determination number, which is required by Accounts Payable if partner compensation is provided via gift cards.

What should you consider when preparing an NHSR IRB application?

- Who you want to partner with or engage in your activities
- What activities you plan to pursue and whether they qualify as NHSR activities
- Whether the title of your application reflects the non-research nature of the project
- If the language in your application aligns with your engagement goals (e.g., using terms like "information" instead of "data," "project" instead of "study," "review" instead of "analyze")

Sample Applications

The following are examples of NHSR IRB applications for various engagement activities qualifying as NHSR:

- Conducting community feedback session(s)
- Creating a Community Advisory Board (CAB)

Frequently Asked Questions

Question	Answer
**When should I expect to hear back from the IRB?**	The turnaround time is typically shorter for NHSR reviews than for HSR full-board or expedited reviews; you should expect to hear back from the IRB within a few days.
**Why did the IRB determine my activity to be HSR and not NHSR?**	It's possible that the phrasing used in the application did not align with engagement framing/goals. See application tips above for more information.<br><br>If the IRB classifies the engagement activity as research: 1) review the application again and revise to better frame your language, 2) re-evaluate whether your activities are, in fact, engagement (if they are research, then a HSR determination is a better fit), 3) contact PaCER for suggestions/feedback, and/or 4) contact UNC's IRB.
**Within the IRB application, how should I respond to the "NHSR Activities" section?**	For "NHSR Activities," we recommend selecting 'Other' and describing the activity as engagement within the textbox. For example, "Our project would be best described as community engagement (engaging community partners to provide input or feedback on the planning, design, conduct, and/or dissemination of research)."

Resources

Useful Links:

- IRB and the Office of Human Research Ethics website
- IRB Online Submission Guide
- One UNC Clinical Research website (for guidance, resources, support services, etc.)
- UNC SOP 501: Human Subjects Research Determination
  - Related: UNC SOP 6001: Definitions
- UNC guidance on gift card payments for participants

Questions? Contact:

- General IRB Questions: irb_questions@unc.edu
- IRB Submissions: IRBIS@unc.edu
- NC TraCS PaCER Program: Request a consultation
