# Supplementary material for: Community feedback sessions: An adaptation of the community engagement studio model to enhance scalability
Source: J Clin Transl Sci. 2026 May 6;10(1):e91. doi: 10.1017/cts.2026.10745 (PMC13237187; doi:10.1017/cts.2026.10745)
Supplement: Frank et al. supplementary material 5 — Frank et al. supplementary material [file S2059866126107456sup005.pdf]

NC TraCS Community Feedback Session (CFS) Service
Guidance on CFS Recruitment and Scheduling Process

General Recruitment Process

Research teams who have contracted with the NC TraCS CFS service have found this general process to be helpful when conducting feedback session recruitment and scheduling:

1. Develop an online screener (e.g., Qualtrics, REDCap, etc.) to assess eligibility and availability of potential feedback session participants. Several potential dates/times that work for both the research team and the NC TraCS team must be identified in advance and included as options on the screener. NC TraCS staff will place holds on their calendars.
2. Develop plain language for a brief recruitment email and/or flyer and include a link and/or QR code to the online screener.
3. Share the recruitment email and/or flyer with your networks and within the communities and/or target populations you wish to engage in your feedback session. *Recruitment avenues that have worked well for others include: clinical/departmental networks; patient networks; patient and family advisory councils; support groups or social media groups run by advocacy organizations or clinics; local chapters of national associations; community-based organizations; and reaching out to a list of past study participants. We can also share your recruitment materials with our network of patient and community partners.*
4. Monitor the online screener and review responses. Identify a date and time when 4-8 people are available to attend a feedback session. *As you review screener responses, consider how you will be including a diverse group of people & perspectives in your conversation.*
5. Once a date/time for the session is finalized, let the NC TraCS team know so we can release the other calendar holds and create a Zoom link for the session.
6. Email individuals to formally invite them to join the feedback session. Share the chosen date/time of the session, ask them to reply to the email to confirm whether they are still available to attend, and let them know that you will be sending a calendar invitation shortly. *It can be helpful to invite 1-2 extra people to the session to account for any last-minute cancellations or no shows.*
7. Create a calendar invitation for the feedback session and send it to all confirmed participants and NC TraCS staff as soon as possible. Ask participants to "Accept" the calendar invitation to re-confirm their attendance.
8. Two to five days before the feedback session – send a reminder email to all confirmed participants. Update the calendar invitation with Zoom information for the meeting if this has not be done already (Zoom link to be created by NC TraCS and shared with your team). Include the 1-Page CFS Information Sheet (prepared by NC TraCS) in your reminder email and attach it to the calendar invitation. Ask participants to review the Information Sheet prior to the session, which will contain additional information about the feedback session topic, how the session will be structured, what to expect, and how to prepare.
9. Send a final reminder email a few hours before the feedback session.

Online Screener Tips

Screeners can help ensure that the experiences and perspectives of those who participate in the feedback session are relevant to your research area and the topic of the session, and can facilitate session scheduling. CFS screeners typically ask for the following information:

1. Name
2. Contact information (email, phone, contact preference)
3. Availability
4. Project-specific or session-specific questions
5. Demographics (e.g., race, ethnicity, gender, age range)

Research teams that have conducted online or social media-based recruitment for feedback sessions have sometimes encountered issues with bots or “insincere participants” completing their screeners. Here are a few strategies that have helped teams mitigate these issues:

1. Enable “bot protection” or reCAPTCHA security settings in survey software.
2. Monitor for signs of spam activities (e.g., mismatched IP address/location, unusually short survey completion time, etc.).
3. Utilize Response Type Validation settings (e.g., enabling this setting for an email field will require all responses to be in email format).
4. Add a hidden question to the screener – this question and any potential responses can be viewed on a submitted screener or in a data export, but it will be hidden for screener respondents. If you see that someone has answered the hidden question, then the response is likely coming from a bot.
5. Do not include compensation amount in initial recruitment materials (e.g., include something like “participants will receive compensation for their time”).
6. Include 1-2 open-ended questions in the screener (e.g., “Please briefly explain (1-4 sentences) why you are interested in participating in the feedback session.” “Please briefly tell us (1-4 sentences) why this topic is important to you.”).
7. If necessary, you can double-screen potential participants (e.g., schedule short, 10-15-minute calls to verify screener information and confirm fit for the session).

Example Community Feedback Session Screener

You can view an example of a community feedback session screener programmed into Qualtrics through this link: [Example CFS Screener]

To view a PDF copy of the example screener, use this link: [Example CFS Screener PDF]
