# Supplementary material for: Community feedback sessions: An adaptation of the community engagement studio model to enhance scalability
Source: J Clin Transl Sci. 2026 May 6;10(1):e91. doi: 10.1017/cts.2026.10745 (PMC13237187; doi:10.1017/cts.2026.10745)
Supplement: Frank et al. supplementary material 6 — Frank et al. supplementary material [file S2059866126107456sup006.pdf]

Feedback Session Evaluation Survey

Thank you for participating in the feedback session! Please take 2-3 minutes to complete this brief evaluation survey. The purpose of this survey is to gain additional feedback about the session and help improve future sessions. Your feedback is anonymous and aggregated responses will be shared with the research team and facilitation team.

If you have any questions, please contact simone_frank@med.unc.edu.

Survey completion date

____

What did you like about participating in the feedback session?

How can future feedback sessions be improved?

In what ways, if any, did the feedback session affect your feelings about or understanding of research?

What contributions did you make to the research project during the feedback session? Please check all that apply.

- ☐ Increased researcher understanding of your point of view or experiences
- ☐ Provided feedback on how practical or doable the project would be
- ☐ Provided feedback on the importance of the study topic
- ☐ Shared ideas on recruiting research participants
- ☐ Shared ideas on how to inform patients, providers, or community members about the project
- ☐ Shared ideas on how to use results of project to benefit the community
- ☐ Other

What other contributions did you make?

____

Please rate your agreement or disagreement with the following statements:

	Strongly disagree	Disagree	Neutral	Agree	Strongly agree
The feedback session was worth my time.	○	○	○	○	○
The researcher's presentation gave me enough information to provide appropriate feedback.	○	○	○	○	○
I felt comfortable sharing my thoughts and ideas.	○	○	○	○	○

Would you participate in a feedback session like this again?

◯ Definitely yes
◯ Probably yes
◯ Not sure
◯ Probably not
◯ Definitely not

You indicated that you may not participate in a feedback session like this again. Can you tell us why?

____

Please answer the following questions about you. These questions are optional but will help us make sure these feedback sessions are inclusive and welcoming to all.

Please select all the groups that describe you.

☐ Patient, caregiver, or community member
☐ Healthcare provider
☐ Other healthcare worker or clinical staff
☐ Researcher or research staff

What is your age?

◯ 18-24
◯ 25-34
◯ 35-44
◯ 45-54
◯ 55-64
◯ Over 65

What is your gender?

◯ Woman
◯ Man
◯ Non-binary
◯ Prefer not to say
◯ Prefer to self-describe

Please describe your gender identity

____

What is your race? (select all that apply)

☐ African American/Black
☐ Asian or Pacific Islander
☐ American Indian/Alaska Native
☐ White/Caucasian
☐ Prefer to self-describe

Please describe your race

____

Are you Hispanic or Latino/a?

◯ Yes
◯ No

Is there anything else you'd like to share?
