# Supplementary material for: Community feedback sessions: An adaptation of the community engagement studio model to enhance scalability
Source: J Clin Transl Sci. 2026 May 6;10(1):e91. doi: 10.1017/cts.2026.10745 (PMC13237187; doi:10.1017/cts.2026.10745)
Supplement: Frank et al. supplementary material 7 — Frank et al. supplementary material [file S2059866126107456sup007.pdf]

Evaluation Survey - NC TraCS Engaged Research Services

Please take 5-10 minutes to complete this brief evaluation survey. The purpose of this survey is to find out how useful you found the engagement services you received from NC TraCS (e.g., community feedback session, data party, engagement meeting, coaching) and to determine how we can improve future services offered by NC TraCS.

This survey refers to: [project_title].

If you have any questions, please contact the NC TraCS staff member with whom you have been working or contact us at our general inbox at engagement.nctracs@unc.edu.

Did the NC TraCS engaged research services you received for this project meet your expectations?

◯ Yes
◯ Somewhat
◯ No

You indicated that your expectations were not completely met. In what ways were your expectations not met? __________________________________

What was helpful about the engagement session(s) supported by NC TraCS?

How can future engagement sessions supported by NC TraCS be improved?

What was helpful about the engagement coaching services you received from NC TraCS?

How can the engagement coaching services offered by NC TraCS be improved?

Please rate the quality of the NC TraCS engaged research services you received in the following areas:

	Extremely poor	Poor	Neutral	Good	Excellent	Not applicable
Overall service	◯	◯	◯	◯	◯	◯
Ease of initiating support (e.g., negotiating MOU and getting started)	◯	◯	◯	◯	◯	◯

Communication with your team throughout the process	◯	◯	◯	◯	◯	◯
Guidance, resources, and information provided by our team	◯	◯	◯	◯	◯	◯
Development and/or review of session materials and content (e.g., discussion prompts, presentations, guides, agendas, activities, etc.)	◯	◯	◯	◯	◯	◯
Session facilitation	◯	◯	◯	◯	◯	◯
Final deliverable(s) (e.g. key takeaway summary)	◯	◯	◯	◯	◯	◯

Would you recommend the engaged research services offered by NC TraCS to your colleagues?

◯ Definitely yes
◯ Probably yes
◯ Not sure
◯ Probably not
◯ Definitely not

You indicated that you may not recommend the engaged research services offered by NC TraCS to your colleagues. Could you tell us why? ______________________

If NC TraCS' engaged research services did not exist, which of the following would be true?

◯ I could have readily found an affordable/free alternative
◯ I could have gotten the service, but it would have been harder/more expensive
◯ I would have needed to redesign my study or approach
◯ I would not have been able to proceed if this service were not available
◯ Other

Please describe your response above: ______________________

May we add your project's engagement materials, such as facilitation guides, presentations, and recruitment flyers, to our repository of resources?

Materials in this repository may be shared with other teams who are conducting engagement activities related to similar topics to help inform the development of their own materials.

◯ Yes
◯ No
◯ Only some materials

Please list the materials we can add to our repository: ______________________

Is there anything else you'd like to share?
