# Supplementary material for: Community feedback sessions: An adaptation of the community engagement studio model to enhance scalability
Source: J Clin Transl Sci. 2026 May 6;10(1):e91. doi: 10.1017/cts.2026.10745 (PMC13237187; doi:10.1017/cts.2026.10745)
Supplement: Frank et al. supplementary material 8 — Frank et al. supplementary material [file S2059866126107456sup008.pdf]

Follow-Up Survey - NC TraCS Engaged Research Services

Please take 5-10 minutes to complete this follow-up evaluation survey. The purpose of this survey is to find out how the engagement services you receieved from NC TraCS (e.g., community feedback session, data party, engagement meeting, coaching) impacted your project.

This survey refers to: [project_title]

If you have any questions, please contact the NC TraCS staff member with whom you have been working or email us at engagement.nctracs@unc.edu

---

In what ways were the engagement session(s) supported by NC TraCS beneficial to your project?

How, if at all, did your research project, plans, or materials change as a result of the feedback you received in the engagement session(s) supported by NC TraCS?

In what ways was the engagement coaching provided by NC TraCS beneficial to your project?

How, if at all, did your research project, plans, or materials change as a result of the engagement coaching you received from NC TraCS?

What challenges, if any, did you face in incorporating the feedback you received in the engagement session(s) into your project?

In what ways, if any, did the engagement session(s) affect your team's understanding of community engagement in research or ability to engage community, patient, or other partners in research?

In what ways, if any, did the engagement coaching provided by NC TraCS affect your team's understanding of community engagement in research or ability to engage community, patient, or other partners in research?

Did you maintain connections and/or continue collaborations with any of the attendees who participated in your engagement session(s)?

◯ Yes
◯ No

Please describe the connections and/or continue collaborations you've maintained with attendees who participated in your engagement session(s): ____

Which outcomes have resulted, if any, from the NC TraCS engaged research services you received? (Select all that apply)
(Select all that apply)

☐ Manuscripts (submitted, accepted, or published)
☐ Conference presentations/posters
☐ Other publications (white papers, etc.)
☐ Grants (submitted and/or funded)
☐ Other outcomes

List citations of submitted, accepted, or published manuscripts related the NC TraCS engaged research services you received.

List citations of conference posters/presentations related to the NC TraCS engaged research services you received.

List and describe other publications related to the NC TraCS engaged research services you received.

List grants submitted/funded related to the NC TraCS engaged research services you received.

List other outcomes related to the NC TraCS engaged research services you received.

Is there anything else you'd like to share?
